# The Benzoates in the Treatment of Tuberculosis

**Published:** 1880-02

**Authors:** 


The Benzoates in the Treatment of Tuberculosis.—A correspondent of the *Cincinnati Lancet and Clinic*, gives the following important views on tuberculosis: In an open letter addressed to the editor of the *Allg. Wein. Med. Zeitung*, No. 1, 1880, Prof. Klebs, of Prague, expresses the present status of his results as to the effects of the benzoates of soda and magnesia in the treatment of tuberculosis in the following statements:

1. The tuberculous miliary eruptions can, by the internal administration of the benzoates, especially of the benzoate of magnesia, be brought to a stand still and to resorption, even in such cases in which further septic infection or marasmus in a high degree have induced a steady advance of the disease. The author maintains that positive proof of this fact is furnished by anatomical investigations, especially of the larynx and kidneys, while the proof on the part of the lungs and intestines, though not demonstrable with the same kind of evidence, is rendered in the highest degree probable.

2. Where the apices of the lungs have not been affected too long, even though attended with high or hectic fever, and with hemorrhages of the lungs, the inhalation alone of the benzoate of soda, or the insufflation and simultaneous internal administration of the benzoate of magnesia will bring about a permanent reduction of the fever, cessation of the catarrhal manifestations, and increase of the body weight to a considerable degree. As in these cases a previous internal administration of quinine had not affected the fever or checked the steady decrease in the body-weight, the good effects of the benzoates were put beyond dispute.

3. There is a certain number of cases affected to the same degree, which resist the influence of this treatment. And there are cases which cannot be ascribed to the advanced marasmus. It is probable that there is in these cases continuous destruction of lung tissue, as from obstinate catarrhal and pneumonic processes extending from destructible cavities. Sometimes we may, in even these cases, break the fever by very large doses of the benzoate of magnesia (one to the thousand of the body weight), after salicylic acid had produced only temporary effects and had then to be ceased on account of the intolerance of the stomach.

In very obstinate catarrhal conditions depending upon, or extending from cavities, catarrhs which are continually recurring are pyretic, or are sometimes unattended with fever, we get the best results, after failure with all expectorant remedies (from the use of which there is never any permanent effects), from pilocarpin. And in these cases the decoction of guiac may be used with advantage. These two agents were used with great efficacy by Max Schuller in animals artificially infected with tuberculosis.

5. If cavities be thoroughly purified by this treatment, so that the sputum no longer shows micrococci, the improvement remains permanent. It is true that light febrile and catarrhal conditions are wont to recur from time to time; but do not involve any loss of the body weight.

6. Finally, there are cases enough in which extensive infiltration of the lungs, great cavities, wide-spread cheesy infiltration of the lymph glands, advanced intestinal and laryngeal tuberculosis render every treatment nil. In these cases the cavities must be entered directly and relieved of their contents, as all experience shows that the infection, especially the septic infection, proceeds from the contents of the cavities.

7. It is very remarkable that of twelve cases which ended fatally, under the above mentioned treatment, there was never a trace of amyloid degeneration, and this in face of the fact that there was profuse suppuration and extreme marasmus.

If then Klebs will not and cannot claim to have discovered a cure for tuberculosis, as people only, but physicians never say, surely no man of science would ever use such an expression, he still maintains that *with this method first used by him, a great number of cases can be brought to a stand still, and to ultimate, absolute, permanent cure.* At least this is true of a number of his cases in which the good result has remained for from three to four years. It is certainly essential that every relapse, every catarrh, every complication, shall be subjected to immediate, energetic, antimykotic treatment. But it is just here that the negligence or indolence of patients so often bring to nought the painstaking care of the conscientious physician.—*Cincinnati Lancet and Clinic.*

